# Recent Advances in Thermoplastic Starch (TPS) and Biodegradable Polyester Blends: A Review of Compatibilization Strategies and Bioactive Functionalities

**DOI:** 10.3390/polym18020289

**Published:** 2026-01-21

**Authors:** Elizabeth Moreno-Bohorquez, Mary Judith Arias-Tapia, Andrés F. Jaramillo

**Affiliations:** 1 Chemical Engineering Program, School of Engineering, Universidad Tecnológica de Bolívar, Parque Industrial y Tecnológico Carlos Vélez Pombo km 1 Vía Turbaco, Cartagena 130010, Colombia; morenoe@utb.edu.co; 2 Departamento de Ingeniería Mecánica, Universidad de Córdoba, Cr 6 #76-103, Montería 230002, Colombia; 3 Department of Mechanical Engineering, Universidad de La Frontera, 01145 Francisco Salazar, Temuco 4780000, Chile

**Keywords:** starch-based biopolymers, polyester blends, bioactive additives, natural compatibilizers, interfacial adhesion

## Abstract

Thermoplastic starch (TPS) blended with biodegradable polyesters such as polyhydroxybutyrate (PHB), polylactic acid (PLA), polybutylene succinate (PBS), and polycaprolactone (PCL) represents a promising route toward sustainable alternatives to petroleum-based plastics. TPS offers advantages related to abundance, low cost, and biodegradability, while polyesters provide improved mechanical strength, thermal stability, and barrier performance. However, the intrinsic incompatibility between hydrophilic TPS and hydrophobic polyesters typically leads to immiscible systems with poor interfacial adhesion and limited performance. This review critically examines recent advances in the development of TPS/polyester blends, with emphasis on compatibilization strategies based on chemical modification, natural and synthetic compatibilizers, bio-based additives, and reinforcing agents. Particular attention is given to the role of organic acids, essential oils, phenolic compounds, nanofillers, and natural reinforcements in controlling morphology, crystallinity, interfacial interactions, and thermal–mechanical behavior. In addition, the contribution of bioactive additives to antimicrobial and antioxidant functionality is discussed as an emerging multifunctional feature of some TPS/polyester systems. Finally, current limitations related to long-term stability, scalability, and life cycle assessment are highlighted, identifying key challenges and future research directions for the development of advanced biodegradable materials with tailored properties.

## 1. Introduction

The high dependence on conventional plastics has led to a massive growth in their production [1], as these materials offer a wide range of properties that are difficult to replicate in alternatives [2]. Approximately 45% of all plastics are used exclusively for food packaging [3]. However, their improper disposal practices have led to significant environmental contamination, due to their non-biodegradable [4] and recalcitrant nature [5]. Between the 1950s and 2015, about 6.3B tons of plastic were produced, of which only 21% have been managed by recycling or incineration [6]. Production continued to rise, reaching 359M tons in 2018 [7]. In addition, COVID-19 intensified plastic waste generation due to the massive use of disposable items [2].

This challenge is particularly critical in food packaging, where materials require resistance and flexibility, typically provided by petroleum-derived polymers [8]. These materials also pose risks to human health, due to the release of chemical and microplastics [9], which have been associated with cardiovascular diseases [10], reproductive disorders [11], and cancer [12]. These problems have led to the search for more sustainable and biodegradable alternatives [13], which can biodegrade more rapidly in natural environments [14], though early bioplastics often exhibited high production costs and inferior mechanical [15].

Starch is a low-cost [4], abundant [16], and fully biodegradable polysaccharide [1], composed of amylose and amylopectin [17]. Amylose consists of a linear chain with α-(1 → 4) bonds, while amylopectin has a branched structure, incorporating the same type of bonds as amylose in the main chain and α-(1 → 6) bonds in the branching zones [15]. Its semi-crystalline structure [18] varies among botanical sources such as potato [19], corn [20,21], sago, peach [5], cassava [14,22], yam [23,24,25], wheat [26,27], sweet potato [28], mango seeds [29], banana, rice [30], jackfruit seed [31], etc. However, the abundance of -OH groups renders starch highly hydrophilic [15], leading to moisture sensitivity [5] and limited mechanical performance [17,18].

Starch can be transformed into thermoplastic starch (TPS) when processed with plasticizers, heat, and shear [5], and modified through processes such as esterification [32], hydrolysis [33], crosslinking [34], and other chemical reactions [16]. To further enhance TPS properties, it is commonly blended with biopolyesters such as PHB, PLA, etc. [35]. These polyesters have become widely used due to their biodegradability [36], stiffness, barrier properties [37], thermal stability, and biocompatibility [38,39,40]. However, their production cost is relatively high [41], so it is an attractive option to blend them with other lower-cost polymers, such as TPS, to obtain more economical and environmentally friendly materials. Among these biopolyesters, polymers like PLA, PCL, and other PHAs show great potential in various applications, such as the packaging sector [42], medicine [43], etc., broadening the spectrum of sustainable alternatives.

In recent years, there has been an increasing interest in the use of naturally occurring agents, such as essential oils and organic acids, as they serve not only as compatibilizers in polymeric blends but also impart antimicrobial, antioxidant, or hydrophobic properties [44,45,46,47]. Such characteristics enable the development of more functional and value-added materials for applications in the food sector.

It should be noted that the TPS/polyester blends discussed in this review are reported in different physical forms depending on the processing method and target application. However, thin films are the most frequently investigated form, particularly in studies addressing morphology, barrier performance, and functional properties.

Considering this, the aim of this review is to critically analyze recent advances in TPS/biopolyester blends, with a particular emphasis on compatibilization strategies involving natural and synthetic agents, including chemical modifiers, reactive compatibilizers, bio-based additives, and reinforcements. This review discusses how these approaches influence interfacial interactions, morphology, and thermal–mechanical performance, as well as the emergence of bioactive functionalities, providing a comprehensive framework for the design of sustainable polymeric materials.

## 2. Thermoplastic Starch and Biodegradable Polyesters: Molecular Features, Sources, and Processing Approaches

**Figure 1:**
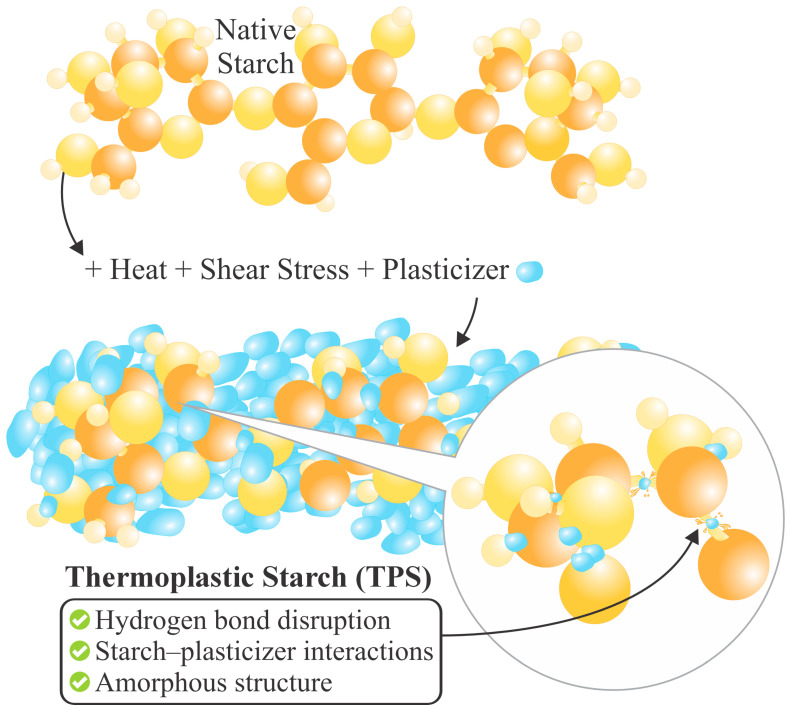
Schematic representation of native starch plasticization under thermo-mechanical treatment and plasticizer addition, leading to TPS formation.

TPS is a material produced from native starch (NS) plasticization [5]. By itself, NS cannot be called a thermoplastic [48], since the intermolecular forces among the -OH groups are very strong. However, the addition of a plasticizer, under conditions of heat and constant stress, can yield moldable behavior akin to those of a thermoplastic polymer [20]. A plasticizer is a compound added to a polymer to provide flexibility under conditions higher than the initial ones [49]. Various substances have been used for this purpose, such as urea, acetamides, sugars [5], glycerol [21], sorbitol, formamide [50], citric acid, polyethylene glycol [51], propylene glycol, xylitol, mannitol [52], glycerol monostearate [27,53], ethanolamine [54], maleic anhydride [14], water, etc. Vegetable and essential oils have also been employed, which can reduce the thermal degradation of NS and improve the processing conditions by lubrication [5]. The NS plasticization process is illustrated in Figure 1.

**Figure 2:**
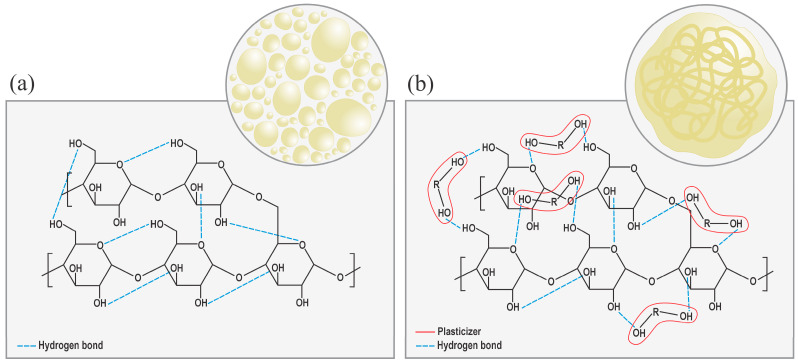
(**a**) Native starch structure with intermolecular hydrogen bonds. (**b**) Plasticizer molecules disrupting starch–starch bonds and promoting starch–plasticizer interactions, yielding an amorphous TPS structure.

Under specified thermo-mechanical conditions, adding plasticizing agents results in the plasticizer molecules interspersing among the starch molecules, thereby disrupting a large part of the -OH bonds (see Figure 1) and reducing the intermolecular forces among them [49]. So, the interrelation between them would no longer be NS–NS, but NS–plasticizer [5], which transforms the NS semi-crystalline structure into an amorphous one, which is characteristic of TPS, as shown in Figure 2 [17]. This transition lowers the glass transition temperature (T_g_) [52], melting point (T_m_), and brittleness, while enhancing flexibility [48]. The plasticization process can be internal (when modifications are made to the chemical structure) or external (when the plasticizing agents do not produce new bonds) [52]. Therefore, it is not only the starch source alone that influences the ultimate material properties [55]; rather, the processing conditions, environment, and type of plasticizer also influence the mitigation of retrogradation [5]. Among the most used methods for TPS production are compression molding [56], casting, and extrusion [13,48]. Extrusion is particularly efficient due to the operational parameters and equipment design, which promote crystalline disruption and facilitate the production of a more homogeneous and structurally coherent material [48].

**Figure 3:**
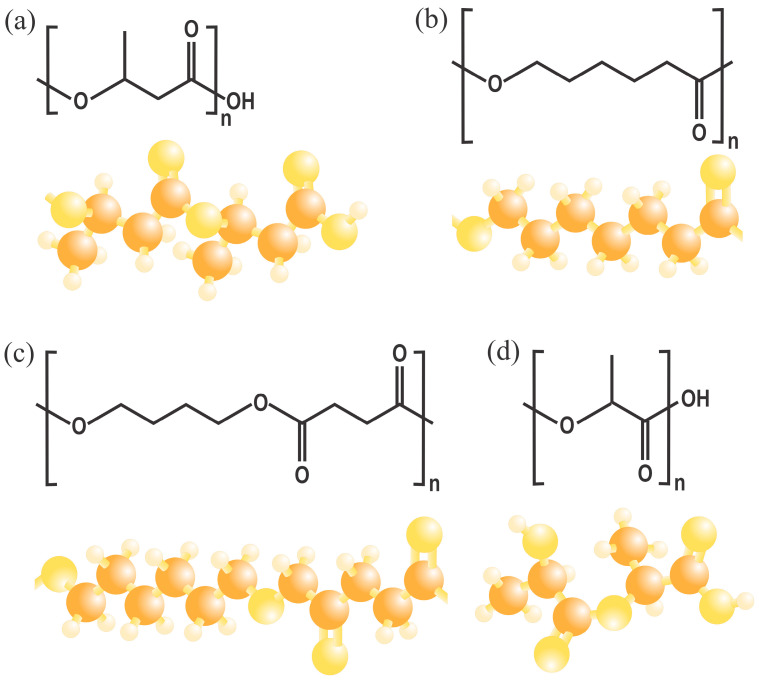
Molecular structures of representative biodegradable polyesters: (**a**) poly(3-hydroxybutyrate) (PHB), (**b**) polycaprolactone (PCL), (**c**) poly(butylene succinate) (PBS), and (**d**) polylactic acid (PLA).

Poly(3-hydroxybutyrate) (PHB) is a biodegradable polyester [57]. It has been the most extensively studied compound of its kind [58] and belongs to the polyhydroxyalkanoate (PHA) family. It is a succession of 3-hydroxybutyrate monomers, consisting of methyl, carboxyl, and ester groups [59,60], as illustrated in Figure 3. This composition results in similar properties to those of some fossil-based plastics [60]. The production costs of this material depend on several factors, including cultivation methods, origin, and the processes used [58]. PHB can be extracted from various sources, such as shells, feathers, and even organic waste containing fish, oil, fruits, vegetables, dairy, rice, etc. [38]. There are even microorganisms (such as *Ralstoniaeutropha*) that can synthesize this type of material [61], as it is usually agglomerated in their cell membranes, reaching in some cases up to a little more than 80% by weight of the cell; therefore, the structure of this material can have certain variations, depending on the different classes of bacteria [58,62]. Its production is a response mechanism of certain microorganisms to stress, usually produced by nutrient restriction during cultivation [63]. There are other methods to obtain PHB, which involve the use of surfactants for chemical digestion, solvents (usually organic) for extraction, and fermentation. However, these tend to be more contaminating than the microorganism synthesis method [39,40].

PLA is a biodegradable aliphatic polyester obtained from lactic acid by polycondensation from raw materials such as corn starch [42]. Its molecular structure is illustrated in Figure 3, which imparts a modulable crystallinity, making it possible to adjust its T_g_ and T_m_ for applications in packaging [64] and 3D printing [65], among other things. PLA can achieve transparency and stiffness comparable to that of some conventional polystyrenes, and it also possesses UV barrier properties [66]. However, it is a relatively brittle material with slow crystallization kinetics, which means that additives must be used to improve its mechanical properties, industrial productivity [67], and, in some cases, its biodegradability, since it must be subjected to industrial composting conditions to be completely degraded [68].

Another biodegradable polymer is PBS, which is synthesized by polycondensation of the monomers succinic acid and 1,4-butanediol, both obtained by fermentation [69]. In addition, it has a T_m_ close to 115 °C and a T_g_ of −22 °C [70], which gives it similar properties to polypropylene (PP) [71]. It also exhibits good processability by both extrusion and blow molding, which favors its use in agricultural applications [72]. However, in recent years, efforts have focused on developing polymeric blends to accelerate their degradation process and reduce high production costs [73]. PCL is a material produced by the ring opening of ε-caprolactone; its chemical structure is shown in Figure 3. It is a polymer with high flexibility and shelf life before degradation due to its low T_g_ and T_m_ [74], which makes it attractive for pharmaceutical applications.

### 2.1. Application Constraints and Processing Challenges

Polymers such as TPS can be produced at low cost, feature barrier properties, and are 100% biodegradable and compostable due to their natural occurrence [13,15,17,48,75]. PHB, being a biodegradable polymer, also poses inherent challenges that limit its applicability. Its thermal resistance is relatively low (See Table 1), constraining its use in applications that require stability at high temperatures. It is markedly more brittle than both biodegradable and synthetic polymers [37]; moreover, it is crystalline (up to 70%), which contributes to its brittle nature and susceptibility to fracture under high mechanical loads. Conversely, although PLA is a highly used biopolymer, its performance has limitations, with a T_g_ around 60 °C (Table 1) [76], which makes it necessary to exclude applications requiring high temperatures. Additionally, this material has a slow crystallization mechanism, which hinders rapid molding and, in some cases, prevents achieving the stiffness needed for high-performance packaging [77]. Being a highly stiff material, the lack of plastic deformation makes it prone to fracturing on impact [78].

PBS, in contrast, is more ductile than PLA and offers good melt processability due to its relatively low T_m_ (115 °C). However, this also limits its application near that temperature threshold [69]. Additionally, PBS has high production costs and has the slowest biodegradation rate within this group, due to limited permeability to oxygen and water vapor [89]. On the other hand, PCL stands out for its flexibility and compatibility with textile materials but is limited in industrial applications by its low T_m_ and T_g_, as shown in Table 1. These properties result in softening, even at elevated ambient temperatures, making it unsuitable for thermally demanding packaging [90]. Furthermore, its elastic modulus and tensile strength are significantly lower than those of other polyesters discussed. Although PCL is inherently biodegradable, its relatively slow decomposition rate restricts its suitability for sustainable biodegradability, and its relatively slow decomposition rate restricts its suitability for sustainable single-use applications [91].

Overall, each of these polyesters presents specific limitations. Recent research has demonstrated that blending them with other components, such as clay, TPS, plasticizers, and lubricants can modify their intrinsic properties, enabling the development of composites that combine the strengths of each constituent. This strategy broadens their potential application across multiple fields [56].

### 2.2. Functional Advantages and Potential Applications

Similarly, biodegradable polyesters have qualities that make them functional alternatives to synthetic plastics. PHB is a high-molar mass polymer [92], and has a T_m_ very close to PLA [93,94], ensuring that the elements manufactured with this material keep their original shape, even up to 132 °C [95]. It has antifungal properties [37], and is considered non-toxic [96], insoluble in water, piezoelectric, and more hydrophobic than starch [58]. Moreover, PHB can be chemically modified during processing through interaction with its functional groups or via reactions such as chlorination, hydroxylation, and methylation [97].

From an environmental standpoint, PHB is associated with a low ecological impact [38] and high biocompatibility, as it can degrade completely in a variety of active environments without leaving residues [37,94]. Its degradation can occur through enzymatic [98], aerobic (approximately 6 weeks), or anaerobic mechanisms, with the latter being the most efficient [60]. Remarkably, PHB’s performance is so comparable to conventional plastics that orange juice packaged in PHB containers has demonstrated similar stability to that achieved in high-density polyethylene (HDPE) bottles, indicating that product acidity is not an issue. Furthermore, its barrier properties regarding water vapor, flavor, odor, and CO_2_ have been found to be on par with those of PET and PVC [59].

PLA combines stiffness and transparency comparable to PET, and provides similar water vapor barrier performance. These features allow it to be used in applications such as thermoformed trays that preserve the freshness of high-moisture fruits, particularly when blended with antimicrobial agents, without compromising product visibility [99]. PBS, on the other hand, offers significantly higher elongation at break compared to other polymers (see Table 1), making it a strong candidate for film production while maintaining mechanical integrity up to approximately 115 °C [81]. In contrast, PCL is characterized by its excellent elongation at break and fast crystallization kinetics, which favor its use in the production of flexible components [100].

Given the inherent properties of TPS, it can be blended with hydrophobic polymers such as PP, PE [26], or the aforementioned biodegradable polyesters to reduce its hydrophilicity and combine the advantageous features of both phases. In recent years, numerous blends have been developed between TPS and polymers such as PP [17,21,101,102], PLA [103,104], PCL [13], PE [75,105], PHBV [106], PVA [107], PBSA [108], and PBS [109], among others.

**Figure 4:**
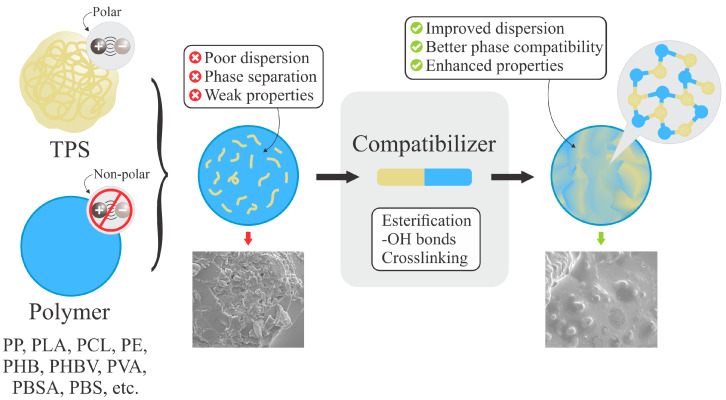
Compatibilization in TPS/polymer blends. Adapted from [110] under the Creative Commons Attribution 4.0 International License (https://creativecommons.org/licenses/by/4.0/, accessed on 5 January 2026).

Nonetheless, since TPS molecules are polar and most hydrophobic polymers are nonpolar, their blends may be heterogeneous when incompatibility arises between components. As illustrated in Figure 4, this incompatibility can be overcome by the use of compatibilizers, which improve phase dispersion and mechanical performance [5]. Such blends not only promote the development of biodegradable alternatives but also contribute to the reduction in fossil-based plastic consumption and greenhouse gas emissions [7].

## 3. Morphological Limitations and Performance Constraints in Non-Compatibilized TPS and Biodegradable Polyester Blends

To improve the biodegradability and crystallinity properties of polyesters, blends with polymeric compounds have been developed recently, such as TPS, which helps to reduce brittleness and improve processability due to their amorphous nature [111]. To produce this type of film, the most used method has been compression molding, which involves placing the previously prepared formulation (usually by internal mixing or extrusion in previous stages) inside a hot mold to be compressed and form films or other solid forms [81]. Table 2 summarizes studies investigating the behavior of TPS blends with biodegradable polyesters and their deviations from the properties of the pure polymer matrices.

Regarding non-compatible TPS/PHB blends, Lai et al. [92] used different types of starch plasticized with glycerol for their blending with PHB, achieving an increase in the mechanical properties compared to the main matrix (TPS); therefore, an appropriate degree of crystallization is crucial if a good performance in the blend is to be achieved. Additionally, the higher the glycerol content, the higher the water absorption. At higher PHB content, the blends exhibited more hydrophobic behavior, due to the molecular nature of PHB.

Thiré et al. [112] found that, in this type of blend, an increase in TPS concentration leads to a decrease in tensile strength, Young’s modulus, and elongation at break, due to the heterogeneous dispersion of starch in the PHB matrix. At the same time, thermal properties did not worsen with the addition of TPS. However, SEM showed phase immiscibility, which can be attributed to the incompatibility in the molecular groups of these two compounds. For TPS/PLA blends, a biphasic morphology has been previously reported with starch particles dispersed in the continuous PLA matrix, typically spherical and with sizes between 1 and 8 μm. This is a result of the low interfacial adhesion between the phases due to their differing polarities, which greatly limits their mechanical properties [113].

In other studies, TPS granules in non-compatibilized blends can reach up to 30 μm, which may act as a stress concentration point and favor premature failure due to crack generation. Given that the tensile strength decreases as TPS increases, the overall mechanical behavior dominates, despite the occasional minor improvement in elongation when compared to pure PLA (10–20%) [114]. Due to the stiffness and weak interfacial adhesion of both phases, non-compatibilized blends exhibited an elongation at break of less than 2% even with somewhat uniform dispersions, demonstrating their intrinsic brittleness. Furthermore, as the system’s TPS content still dominates the degradation rate, this absence of compatibilization has no discernible effect on the system’s degradability [115].

Ternary blends can be obtained when employing additional compounds in TPS/PLA blends. Vanovčanová et al. [116] made PLA/TPS/PHB blends, varying only PHB and PLA contents at a processing temperature profile of 160–190–170 °C, and, considering that PHB can degrade under such conditions, it was demonstrated that the PLA and TPS blend achieved improved performance in terms of processability, since the presence of PLA promotes a redistribution of the plasticizer from TPS toward the polyester-rich domains.

This redistribution reduces the effective exposure of PHB to thermally induced chain scission and enhances the melt stability of the blend. Additionally, it was found that the modifications in the mechanical properties resulted from thermal absorption that restores the molecular mobility lost during sub-T_g_ aging and promotes the relaxation of internal stresses. As mobility is recovered, the amorphous regions undergo structural reorganization, modifying the balance between stiffness and deformability [120].

According to reports, the type of starch employed in TPS/PBS blends may enhance the features of the polymeric blends even in the absence of compatibilization. Non-compatibilized blends made of waxy (WTPS, 0% amylose) and native (NTPS, 26% amylose) starch were assessed by Li et al. (2013) [117], who found that WTPS was easier to plasticize and allowed for better dispersion in PBS, which resulted in improved mechanical properties and higher processability compared to blends containing NTPS. As the PBS content increased, the blends showed higher strength and elongation, but lower stiffness. WTPS also reduced water absorption and improved thermal stability at high TPS contents, due to its more homogeneous morphology, demonstrating that the type of starch has a decisive influence on the properties of these types of blends [117].

Alternatively, non-compatible TPS/PCL blends can form limitedly miscible systems, since PCL usually acts as a continuous phase and TPS as a dispersed phase, even with a high TPS content, due to the higher fluidity of PCL in the blending processes [118]. In some cases, it has been possible to establish a certain level of compatibility promoted by -OH bonds [119], yet the blends remain significantly brittle since the interfacial adhesion is not strong. This means that PCL, although it reduces part of the moisture absorption, fails to prevent the retrogradation process after storage, which is more influenced by the environmental humidity than by the PCL content [118]. Finally, the crystallinity in this type of blend also depends on the starch source used, since the branched structure of amylopectin interferes with the crystalline order of PCL [119].

In general, non-compatibilized TPS/biodegradable polyester blends exhibit biphasic morphologies with poor interfacial adhesion, which adversely affects mechanical integrity and structural stability. Although each polymer contributes valuable attributes, their intrinsic immiscibility remains a major limitation, reinforcing the need for compatibilization strategies to achieve balanced and long-term performance.

## 4. Compatibilization and Reinforcement of TPS/Biopolyester Blends for Enhanced Properties

TPS and biopolyesters are high-potential materials for biodegradable plastics production [5]. For instance, polymers such as PHB degrade completely in both land and marine environments at temperatures from 21 °C, according to the TUV test [121,122]. Although polyesters provide enhanced stiffness and thermal resistance compared to TPS [58], most PHAs still exhibit brittleness and limited impact strength [123]. For this reason, they have been blended with other compounds such as PLA [116], PVA [124], or TPS [92]. However, these systems are often incompatible due to the differences in polarity and molecular structure, since TPS is hydrophilic [75], resulting in phase immiscibility and premature mechanical failure. Consequently, recent research has increasingly focused on compatibilization strategies to improve interfacial adhesion and stabilize TPS/biopolyester blends [122,125].

**Figure 5:**
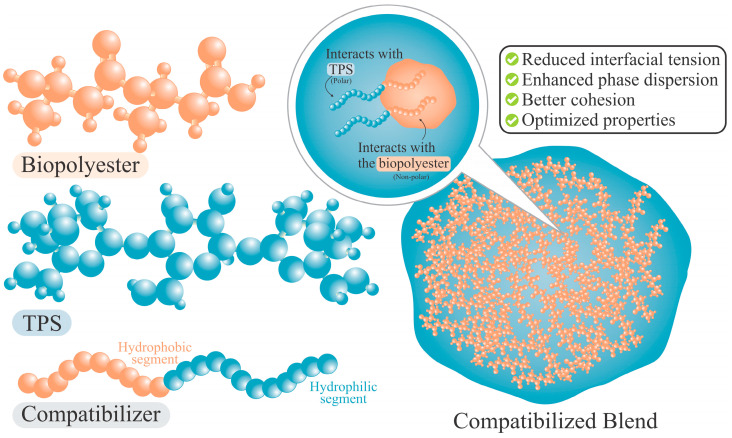
Schematic of compatibilizer action in TPS/biopolyester blends.

Compatibilizers are substances that can be strategically placed on contact surfaces to reduce interfacial tension and achieve improved integration between two or more components. The principle of compatibilization is based on interactions (chemical or physical) occurring among polymer functional groups. The compatibilizer agent has segments that bond or interact with one of the blend phases, while the other segments link to the second one [126], as shown in Figure 5. As a result, the interfacial energy decreases and the TPS polar group is more effectively dispersed within the PHB nonpolar phase, promoting finer phase distribution and enhanced interfacial adhesion [17]. Therefore, a compatible blend is characterized by a uniform distribution of the minority phase within the main matrix, along with a strong adhesion between the two phases. Hence, any applied force can be effectively distributed across all phase components, thereby achieving effective structural integration [102].

As for polyesters, different types of compatibilizers have been employed, which include plasticizers, such as lubricants, nucleation agents [58], or even components to reduce the T_g_ [61]. Owing to the internal structure of polyesters, in some cases, nanofillers can be added to enhance the mechanical properties by penetrating the pore spaces of the matrix and thus improving the interfacial adhesion by providing a more exfoliated surface [75]. Furthermore, the addition of antioxidant components to the blends can stabilize them during processing and increase the viscosity of the matrix for better blending [81].

### 4.1. Compatibilization Using Conventional Agents and Functional Additives

By improving starch dispersion in PHB and water resistance, Liao and Wu [127] developed PHB/starch and PHB crosslinked with acrylic acid blends, resulting in notable improvements over the primary matrix. This was explained by the reaction between the carbonyl groups of the PHB graft and the -OH groups of the starch, which produced ester groups. Additionally, the compatibilized blend exhibited improved tensile strength at breakpoint with a starch content of up to 50 wt.%. A decrease in T_m_ accompanied by an increase in melting enthalpy (ΔH_m_) was observed as the starch content increased. This behavior is linked to the lower melt viscosity of starch compared to PHB, which reduces the temperature required for melting, while simultaneously promoting more efficient packing of crystalline domains, thereby increasing ΔH_m_. As a result, the morphology of the blends was more uniform compared to the non-compatibilized blend.

Similarly, another grafting-related investigation was conducted by Don et al. [124], where PHB was blended with a starch–polyvinyl acetate (PVAc) graft, achieving a grafting efficiency of 12.5% and complete miscibility in all formulations. As a result, an increase in the thermal properties of the material and a reduction in the molecular weight of PHB in processing were evidenced, resulting in a more tenacious PHB and improved interfacial adhesion. However, the compatibilizer used had a low biodegradability, which might negatively affect the blend in terms of degradation.

Florez et al. [128] performed TPS with plasma crosslinked PHB and sulfur hexafluoride (SF_6_) blends by compression molding to evaluate the material compatibilization, which resulted in a homogeneous distribution of PHB within the TPS matrix. The higher PHB content (even when modified) resulted in some agglomerations on the blend surface, which can become stress concentrators and adversely affect not only the interfacial adhesion but also other mechanical properties, thermal stability, crystallinity, and mechanical properties increased significantly compared to the TPS matrix. One exception was the elongation at break, which exhibited a decay. Therefore, this combination of materials offers promising results for applications that do not require high material elasticity.

Regarding TPS/PLA blends, one of the most prominent strategies involves incorporating grafted copolymers, as shown in Table 3. Trinh et al. [129] synthesized a starch copolymer grafted onto PLA by ring-opening polymerization, upon the incorporation of a 2.5–5 wt% in TPS/PLA blends (60/40), a remarkable morphological improvement was achieved, evidenced by a reduction in the size of PLA spherulites, greater homogeneity of phases, and significant enhancements in mechanical and barrier properties, with no need for covalent bonds between phases, thus facilitating the recyclability of the material.

On the other hand, the use of citric acid (CA) as a compatibilizer and plasticizer in TPS/PLA blends has also been explored; this one can promote the partial esterification of starch and enhance its interaction with the PLA matrix. By adding a 5% CA ratio, it is possible to achieve a balance between thermal, mechanical, and barrier properties. This resulted in a higher level of structural fragmentation, which accelerated PLA’s biodegradability and strengthened its potential for sustainable applications because of its non-toxicity and widespread use in food applications [130].

In addition to organic compatibilization strategies, an alternative approach involves the use of organic fillers, like zeolite, combined with orienting procedures, as proposed by Rosa et al. [1]. These fillers offer a property profile that is difficult to achieve with polymeric modifiers alone [2] and can significantly enhance interphase compatibility. By reducing the size of the dispersed TPS phases and improving their dispersion within the PLA matrix, zeolite incorporation leads to positive results of up to 90% in oxygen barrier performance and 100% in tensile strength relative to the uncompatibilized blend. This demonstrates how an inorganic filler, such as zeolite, can effectively improve the properties without compromising the biodegradability of the system.

Reactive chemical modification is one of the most widely used compatibilization techniques for TPS/PBS blends. By adding PBS functionalized with terminal isocyanate groups, miscibility with TPS is significantly improved, with tensile strength up to 10 times that achieved with just 10% compatibilizer. This, in turn, reduces water absorption, increases hydrophobicity, and promotes a more uniform dispersion of PBS in the TPS matrix [132]. Similarly to the TPS/PLA samples, Beluci et al. [133] developed biodegradable TPS/PBS/PBAT films by extrusion, employing CA as a compatibilizer to enhance compatibility, mechanical strength, and opacity without affecting the color. In addition, better interaction of the components and a more ductile and rigid blend were exhibited, demonstrating that CA is useful for this purpose in different types of polyester.

Maleic anhydride and tartaric acid have also proven to be good compatibilizers for TPS/PBS blends in the presence of organotin catalysts, as they promote the formation and grafting of copolymers onto TPS, improving interfacial strength and adhesion, as previously confirmed by Fourier transform infrared spectroscopy (FTIR) [134]. On the other hand, reactive blends have also shown high efficacy in this function. Thajai et al. [135] incorporated chlorhexidine gluconate (CHG) into TPS for blending with PBS and epoxy resin. The reaction between the amino groups of CHG and the epoxies of the resin enhanced interfacial adhesion, resulting in improved mechanical strength, thermal stability, and hydrophobicity, which demonstrates the effectiveness of this blend for developing advanced bioplastics. Collazo-Bigliardi et al. [131] employed PCL with glycidyl methacrylate and maleic anhydride as compatibilizers in TPS blends. While preserving good mechanical integrity, the addition of 5% PCLG enhanced interfacial adhesion and dramatically decreased oxygen and vapor permeability. Thus, PCL can decrease matrix stiffness and increase elongation. Comparable qualities to LDPE were provided by PLA/TPS/PCL formulations, demonstrating the efficacy of PCL as a structural and flexibilizing agent [136].

Polyester inherently presents functional groups capable of interacting with similar segments in other polymeric components, which can facilitate interfacial adhesion in certain blend systems. In contrast, native starch exhibits limited compatibility with hydrophobic matrices. However, though esterification with chemical agents, its structure can be modified by introducing ester groups along the chain, thereby enhancing interfacial adhesion and better miscibility within polyester-based blends [109].

Its capacity to create ester linkages has led to extensive research into CA as a crosslinking agent in various starch matrices. Li et al. [137] examined the impact on wheat starch granules following a heat and moisture treatment after CA esterification. They achieved levels of 71% in both type A and type B granules, demonstrating a significant improvement in the synthesis of thermally resistant starch. The treatment was successful in altering starch crystallinity and increasing the degree of substitution, indicating efficient ester formation. Moreover, SEM showed collapsed granules due to structural modification, suggesting that CA esterification, especially when combined with this type of treatment, not only alters physicochemical properties but also offers thermal stability and digestibility in biodegradable film systems.

Similarly, CA-modified starch nanoparticles have been created that exhibit improved hydrophobicity and thermal stability through the formation of ester groups, as verified by FTIR. This illustrates how well CA works at the nano scale to modify materials to be compatible with hydrophobic ones [138], like polyester. Yang et al. [139] combined CA and oxidized palm oil oligomers to create composite materials based on fiber and starch. CA demonstrated high compatibility by improving interfacial compatibility and mechanical strength by esterification and crosslinking, significantly enhancing modulus and tensile strength with only 0.75% oxidized oil.

CA is also useful as a stabilizing agent in composite systems; its addition to tiger nut starch has been previously tested, obtaining esterification with a high content of resistant starch, decreased swelling, and solubility. This shows a reduction in crystallinity and an increase in humectability, improving its performance in Pickering solutions [140]. In fibrous starch and paper pulp composites, CA has also been shown to act as a crosslinker, by significantly improving the water resistance and mechanical strength of processed sheets [141], thereby reinforcing its potential to alter the processability and compatibility in relevant hybrid systems [142] for PHB–starch blends.

Beyond modifying individual granules, He et al. explored how CA, glycerol, and stearic acid (SA) functioned as plasticizers in TPS blended with PBAT, a polymer similar to PHB in its hydrophobic nature. It was observed that a 30% glycerol composition provided superior dispersion and yield of TPS compared to native starch [143]. SA is a compound that has also been attractive as a compatibilizer due to its ability to impart hydrophobicity and introduce long aliphatic chains into the starch structure through esterification [144,145]. An investigation of starch stearate sintering in aqueous urea/NaOH solutions reported substitution degree values higher than 0.065, lower crystallinity, higher thermal stability, and better emulsifying and moisture barrier properties than native starch [146]. Similarly, stearates were developed using a dry method under atmospheric conditions, in the absence of catalysts or solvents, with regenerated starch as the precursor, achieving a maximum degree of substitution of 0.3. Hence, the increase in this parameter led to improved emulsification capacity, freeze–thaw stability, and reduced moisture adsorption [147]. This suggests that esterification with SA is a scalable and efficient method for blending starch with PHB, where hydrophobic compatibility is essential for achieving phase dispersion and mechanical cohesion.

EA dual-modified cassava starch esters were synthesized by Lozano et al. [148]. In 2024, it was tested as a matrix in composites with microfibrillated cellulose. The results showed that the esterification method increases hydrophobicity and decreases the crystallinity of the phase. High substitution degree values, however, enhance barrier qualities but may also make some blending systems less compatible and more brittle [149]. Although CA and SA are different in structure and effect, both contribute positively to the mechanical and environmental performance of starch films. Thus, their suitability should be chosen based on the target application and process composition, as the benefits provided by each are complementary. CA improves crosslinking and structural rigidity, while SA can improve water resistance and hydrophobicity. This duality implies a potential strategy for the multifunctional compatibilization of starch with hydrophobic polymers [32], as summarized in Table 4.

In addition, it should be noted that, although there is a variety of compounds widely used as natural and synthetic additives, their safety for food contact depends on their chemical composition, concentration, and potential migration [150]. Some studies indicate that certain compounds can migrate into food; therefore, their limits must be validated according to the current regulations applicable to the area in which the material is developed. Thus, specific migration tests are essential to confirm their safe use in food packaging [151].

### 4.2. Phenolic and Oily Compounds as Natural Compatibilizers

If a polymer blend with improved properties is desired, the use of additive compatibilizing agents is essential. However, many of these may be detrimental to human health in applications involving direct contact with these plastics in consumer products, such as packaging. Therefore, promoting natural alternatives that fulfill the same compatibilization functions is necessary [152]. Previous studies have shown that many natural species of botanical origin can accomplish these purposes while extending the shelf life of the products inside [153]. In addition, these compounds have biodegradable properties, are easy to degrade, and are biocompatible, since they come from organic sources, which gives them a high potential as additives in polymeric blends [154].

Some bioactive compounds present in natural sources, such as polyphenols and even essential and conventional oils, have previously been employed as chemical additives [153]. Essential oils are scented products derived from plant sources, usually separated by steam, distillation, or other advanced techniques, and their lipidic nature allows them to be easily separated from the liquid phases without the need to use methods that modify their chemical structure [155]. These can be extracted from various plant spices, such as coriander [156], lavender [157], lemon [158], rosemary [159], mint [160], turmeric [161], eucalyptus [162], cinnamon [163], orange [164], moringa [165], garlic [166], clove [167], thyme [168], verbena [169], sage [170], chamomile [171], citronella [172], tangerine [173], oregano [174], and others.

Phenol-rich oils have been proven to be effective against a variety of pathogens, including resistant bacteria. Their mechanism of action involves altering cell membranes and inhibiting resistance systems, including cell–cell communication and biofilm formation. However, some aspects, such as light, temperature, or atmospheric oxygen, must be considered, as they may be affected due to the high tendency for oxidation reactions [175].

In the last few years, several oils used for addition to polyesters and TPS have been found to modify their properties depending on their extraction source (Table 5). Garcia-Garcia et al. [176] studied the effect of maleinized linseed oil (MLO) and epoxidized fatty acid ester (EFAE) at low concentrations in improving the toughness of PHB in industrial formulations. Tensile strength and elongation at break were enhanced by this addition, with EFAE exhibiting a somewhat higher strength. Additionally, the PHB matrix’s compatibility with these plasticizers reduced porosity, producing a homogeneous material with improved structural continuity. Furthermore, compared to the pure matrix, both components increased the degradation temperature and decreased the T_m_ of PHB, thereby increasing its processing window.

Consequently, another study was also conducted using epoxidized linseed oil (MLO) and soybean oil (ESBO) to enhance the properties of PHB. MLO was found to be more effective than ESBO, particularly at a concentration of 10%, where improvements were evidenced in terms of increased elongation at break and energy absorbed per impact. However, the incorporation of both oils brought an improvement in the thermal stability of PHB through an increase in its degradation temperature, contributing to the optimization of formulations for industrial applications [79,177].

Other types of oil, such as essential oils, have also been used in blends with polyesters, like PHB, where even better properties, including antioxidant and antimicrobial effects, have been found. In research, three different types of essential oils (cinnamon, melaleuca, and citronella) were incorporated into PHB. The citronella–cinnamon and melaleuca–cinnamon oil combinations increased the flexibility of PHB, known to be a highly rigid material, and also inhibited the proliferation of various microorganisms. However, the melaleuca–citronella oil combination had no antimicrobial activity, and the PHB samples using it demonstrated minimal UV radiation transmission. Nevertheless, the addition of essential oils improved the crystallinity and thermal stability of PHB, while decreasing its melting temperature compared to the pure matrix. This particular type of composition has potential applications in the bioactive packaging industry [46].

Cinnamon oil variants, such as cinnamaldehyde, have been utilized as compatibilizing agents in PLA/PHB films, in conjunction with mono-caprylyl glycerate (MCG) and glycerol monolaurate (GML) as plasticizers. As a result, an improvement in the mechanical and active properties of the films was achieved, also exhibiting microbial activity, as demonstrated in an analysis performed with the preservation of a piece of salmon. Additionally, increases in tensile strength and antioxidant properties were observed as a result of the addition of cinnamaldehyde [64]. Albuquerque et al. [179] conducted a study to evaluate the antimicrobial effect of clove oil in PHB and bacterial cellulose blends in acetic acid. This time, such an addition enhanced the hydrophobic properties of the film and facilitated the blending of the components through a compatibilization effect, thereby improving the interactions between the phases. The microbial growth was reduced by up to 65%, while the mechanical and thermal properties showed an improvement, as evidenced by an increase in tensile strength and a higher degradation temperature. Thus, essential oils have multiple applications, and their performance can vary depending on the affinity with the components of the blend.

Another essential oil that is highly investigated is eugenol, which is not only antimicrobial and antioxidant, but also modifies the structure of PHB by processes such as extrusion [184]. A study analyzing the effect of adding eugenol to PHB in degradative processes revealed an antimicrobial oil-dependent behavior present in the matrix, which increased the crystallinity but caused a slight decrease in the mechanical properties. It should be noted that, at the time of degradation, the rate of polymer hydrolysis can vary depending on the degree of molecular disorder [180]. Garrido et al. developed biocomposites by blending TPS and PHB using organically modified montmorillonite (OMMT) as nanofiller and eugenol as a compatibilizing agent. An enhanced exfoliated surface and a sustained degradation temperature were achieved. However, there was a reduction in the T_m_ and elastic modulus. The crystallinity exhibited an increase, and both antifungal and antioxidant activity were evidenced by the effect of eugenol [35].

Finally, an eco-friendly method was suggested by Volpe et al. [183], who reported that used sunflower oil can function as an efficient plasticizer by partially substituting it for glycerin in starch grading. To reduce expenses and advance the circular economy, the addition of this fried oil enhanced TPS’s thermal stability and uniformity when combined with PLA. Although the study focused on PLA, the findings highlight the potential of conventional oils in the compatibilization of starch/polyesters blends, underlining that the ratio of oil to glycerol and the type of oil have a significant influence on the final performance and material properties.

Each essential oil modifies the polymer properties differently, depending on its nature and affinity. Similarly, the mechanism of action varies according to the components that comprise it. Since they all belong to the lipid group, the oxidation process in oils can be summarized in three phases: initiation, propagation, and termination [185].

Triplet oxygen (O_2_) is the ground state form of atmospheric oxygen, which is distinguished by the presence of two unpaired electrons with parallel spins. With a spin quantum number of 1, this is the most stable form of oxygen under normal circumstances. Additionally, unsaturated lipids contain bonds (C=C), which are relatively reactive. However, under standard conditions, the electrons in these bonds are paired in a state known as a singlet, i.e., the electrons are arranged in pairs with opposite spins, which would give a spin quantum number of 0 [186]. According to quantum chemistry, for a reaction to occur without the need for high activation energy, the total spin of the reactants must match the total spin of the products. Given the difference between the spin numbers of triplet oxygen and unsaturated lipids, for a direct reaction to occur, one or more of the unpaired electrons must reverse their spin [187]. These types of reactions are known as “spin forbidden” and are very unlikely to occur, as they require the spins to be reoriented, and this needs additional energy or specific conditions for oxygen to react with lipids [188].

**Figure 6:**
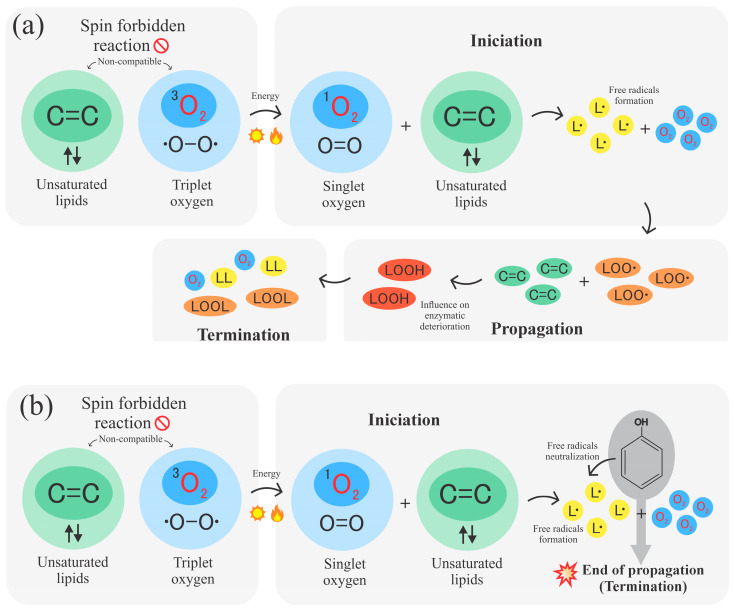
(**a**) Lipid oxidation mechanism through initiation, propagation, and termination. (**b**) Antioxidant activity of phenolic compounds via free radical neutralization.

Such a change in spin can be accomplished by additional energy supply via light, heat, catalysts, or singlet oxygen (^1^O_2_), a more reactive form of oxygen in which two electrons are paired in an excited state. During this state, oxygen can react more easily with lipids, as their spins are compatible, and here is where the initiation process takes place [188]. Subsequently, in the propagation stage, lipids are transformed into free radicals, which react again with the oxygen present in the atmosphere and then form new peroxide radicals. These remove hydrogen atoms from other lipid molecules, forming hydroperoxides, as illustrated in Figure 6a. This process is repetitive, and this mechanism causes the oxidation process to result in autocatalysis. The hydroperoxides formed are unstable compounds that usually decompose, producing a variety of volatile and non-volatile compounds, including radicals. The compounds produced usually influence oxidative enzymatic deterioration. During the termination phase, radicals combine and give rise to non-radical products, and any reaction that prevents propagation or removes free radicals from the system is fundamental to the mechanism of this phase [189].

Oils, particularly the ones containing phenolic compounds, can act as antioxidants due to the presence of hydroxyl (-OH) groups in their structure, which can donate hydrogen atoms to free radicals, thus converting peroxide radicals into less reactive forms, stopping the propagation of the oxidation reaction, as shown in Figure 6b. In addition, these oils can bind to other radicals or react with each other to form non-radical species, contributing to the termination phase of lipid oxidation, where radicals combine and neutralize, effectively halting the oxidation chain [185]. So, theoretically, antioxidants reduce the development of the propagation phase in the oxidation mechanism, thereby shortening the path from initiation to termination.

Oils containing phenolic compounds are usually derived from specific plants. Among the most common phenolic compounds are eugenol [190], cardanol [191,192], cardol [193], thymol [194], and carvacrol [195]. Although widely recognized for their antioxidant and antimicrobial activity, these phenolic structures are also functional groups able to interact with polymer matrices, which is relevant when evaluating their potential roles in blend performance. Other essential oils, such as citrus oils, have a different chemical composition (terpenes), present different chemical compositions, but can still provide antioxidant effects [196].

These phenolic compounds may constitute up to 85% of the total oil composition [185], and their chemical reactivity contributes not only to bioactivity but also to possible interactions with polymer chains. Their natural origin [184] and antibacterial activity [197], in *E. coli*, *S. aureus*, and *Eurotim amstelodami*, among others, make them appealing multifunctional additives [184]. They occur in plants such as thyme, oregano, cashew [198], etc. Particularly, carvacrol, one of the main components of oregano oil [45], and thymol [199] have shown high oxidative and antimicrobial performance [200] due to their phenolic nature [201]. Cardanol and cardol, obtained from cashew nutshell liquid (CNSL) [202], present similar multifunctional characteristics.

Cashew contributes an important source of bioactive compounds [203] and is widespread across tropical regions. Although its chemical composition varies with species and environment [204,205,206,207,208], its shell liquid consistently contains anacardic acid, cardol, and cardanol as major constituents [209,210]. Extraction techniques such as cold pressing and solvent extraction [211] influence the proportion and functionality of these molecules.

Previous studies incorporating oils into polymeric matrices have shown that the antioxidant capacity of the phenolic components strongly dictates the overall activity of the oil [185]. Therefore, understanding their molecular interactions becomes essential when formulating polymer blends [212]. Recent studies revealed that cashew oil and its phenolic derivative cardanol can plasticize starch and serve as alternative modifiers for TPS, even with low or no traditional plasticizer loading [213,214]. This method is especially appealing for enhancing the functionality and processability of starch-based systems, including later blending with hydrophobic polyesters to create biodegradable composites. Its ability to undergo epoxidation or esterification [215,216] further expands its role from a simple plasticizer to a potential reactive modifier capable of promoting phase adhesion.

As a result, TPS modified with CNSL derivatives becomes more hydrophobic and more resistant to moisture [214]. The aromatic ring of cardanol may also provide some thermal and oxidative stability relative to common aliphatic plasticizers [215]. The hydrophobic features of the alkyl side chain [213,214] also better match the hydrophobic nature of polyesters by improving interfacial adhesion and retaining its molecular weight during melt extrusion by scavenging free radicals.

Regarding other oils, Fayyazbakhsh et al. [217] investigated how eucalyptol, limonene, and thymol affect PHB degradation. The results showed that samples containing 3% of these terpenoids slowed down the degradation process, achieving a maximum of 76.9% in 200 days, compared to the degradation of pure PHB, which reached 100% in 198 days. Due to its antibacterial qualities, eucalyptol also provided the greatest mechanical resistance and delayed biodegradation, followed by limonene and thymol. Although these terpenoids primarily act as bioactive modifiers, their influence on crystallinity and T_g_ indicates that they may also contribute indirectly to phase stability in polyester-rich systems.

In some blending processes, better performance has been evidenced in oils that are encapsulated prior to processing, as they are sensitive and can be affected during processes such as extrusion [218]. Additionally, this pretreatment enables the gradual release of the essential oil, which is crucial for maintaining prolonged antioxidant activity over time. It can also be useful to prevent the oil from acting as a plasticizer and weakening the structure of the main matrix [219]. Nanoencapsulation can enhance antimicrobial efficacy. A study performed encapsulation in oils of oregano (*Origanum vulgare*) and thyme (*Thymus capitatus*), with a composition of 73% carvacrol and 44% thymol in a PCL matrix using a nanoprecipitation method, resulting in high encapsulation efficiency and stability. Compared to pure oils, both thyme oil-loaded and oregano oil-loaded nano-capsules achieved higher antimicrobial activity than the oils in their pure state [220].

These components also exhibit a compatibilizing and antimicrobial effect in blends containing nanofillers. Campos-Requena et al. [221] carried out an investigation in which films of TPS nanocomposites with OMMT were prepared using a blend of carvacrol and thymol to create an antimicrobial packaging suitable for fruit preservation by the extrusion method, which exhibited a more exfoliated morphology compared to the main matrix. The mechanical properties increased along with the thermal properties, with a controlled release of the additive components, which decreased by 43%. In addition, it was shown that a combination of carvacrol and thymol could achieve higher antimicrobial activity compared to pure carvacrol, without affecting fruit quality parameters.

Therefore, polymeric blends with these types of compounds as compatibilizers have a high potential, particularly for the development of bioactive packaging for fresh foods [222], since they act as antimicrobial and antioxidant agents, extending their shelf life [223]. However, prior component studies are needed to verify their compatibility. Previous studies have reported that it is essential to have starches with large amorphous regions that facilitate the gelatinization of the starch, which can be achieved with a high amylose composition [56]. Also, further testing, especially on sensory factors and commercial acceptability, can further reinforce this premise [221].

### 4.3. Compatibilization and Property Enhancement Using Nanofillers and Natural Reinforcements

**Figure 7:**
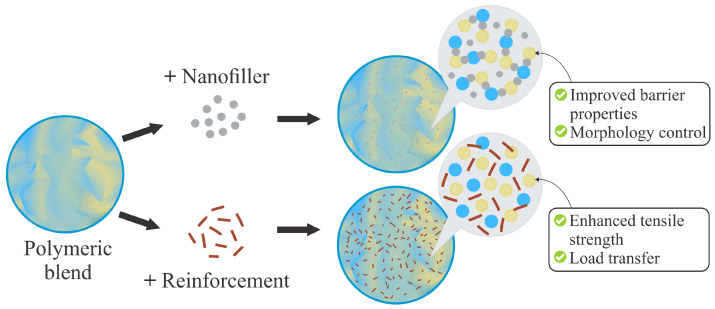
Schematic representation of TPS/biopolyester blends modified with nanofillers/reinforcements.

TPS/biopolyester blends have been widely studied in order to combine sustainability, processability, and functionality. However, the limited compatibility between the phases and the fragility of the resulting systems have motivated the incorporation of natural reinforcements or nanofillers to improve their performance. This general mechanism is illustrated in Figure 7, where the addition of nanofillers or reinforcements promotes barrier and morphological control, as well as enhanced mechanical performance through load transfer.

Materials produced from PHB and TPS with the addition of jute fiber as reinforcement (up to 30 wt%) showed improved thermal stability and reduced water sensitivity [224]. However, the amount of glycerol should be controlled, since a high composition can reduce mechanical properties and increase water sensitivity due to its hydrophilic nature. On the other hand, the addition of fiber increases the material’s capacity to withstand stress, maintaining its strength even after hours of exposure in humid environments.

Nanofillers and essential oils have also been employed as compatibilizers in PHB and TPS blends. Garrido et al. performed blends with OMMT clay and eugenol, resulting in an exfoliated, rough surface and enhanced interfacial adhesion in the blends compared to the PHB matrix, attributed to the migration of the clay layers, which also improved the elastic modulus and the material’s stiffness up to 50 °C. However, the addition of clay produces a slight decrease in the degree of crystallinity, limiting the mobility in the bionanocomposite segments. Thus, this is a good alternative as long as the levels of both clay and TPS are moderated, therefore positioning PHB as a potential sustainable alternative in terms of synthetic polymers involving packaging applications [111,225].

Other studies have also explored the viability of improving the properties of TPS/PLA blends by using fibers to combine the advantages of both polymers. Serra-Parareda et al. [226] employed bleached Kraft fibers, and a 30% ratio increased Young’s modulus over 100% compared to the non-reinforced blend, achieving values of 5.54 GPa. Other types of fibers, like coconut fibers, also improve the interfacial compatibility and stiffness in the system, as evidenced by the formation of hydrogen bonds between the phases and the increase in PLA crystallinity [227]. In addition, the use of nanofillers such as OMMT enhances the properties of TPS/PLA blends, promoting exfoliated structures that improve the tensile strength, impact, and biodegradability of the material [228].

Regarding TPS/PBS, Boonprasith et al. [229] demonstrated that the addition of nanoclays significantly increased the tensile strength and improved the thermal properties, especially when a high proportion of TPS (75%) was used due to the intercalation and exfoliation of the clay in the main matrix. In contrast, the reinforcement mechanisms associated with natural fibers differ from those of inorganic nanoclays, as their efficiency depends primarily on the interfacial compatibility. Thus, various surface treatments such as alkali, silanization, acetylation, etc. have been used, significantly helping to improve stress transfer, leading to an increase in the elastic modulus and tensile strength [230]. On the other hand, for TPS/PCL blends reinforced with sisal-derived cellulose nanocrystals (Whiskers), surface changes in the nanomaterials promoted uniform dispersion and chemical interaction that resulted in an enhancement in crystallinity [231].

These results support the approach of blending biopolyesters with nanofillers and reinforcements to create bio-based materials with characteristics suited to different uses, offering a sustainable substitute for conventional plastics.

## 5. Future Perspectives

TPS blends with biodegradable polyesters such as PBH, PLA, PBS, and PCL have demonstrated promising thermal and mechanical performance. However, their long-term stability and industrial scalability remain unresolved challenges. Although many studies highlight property improvements through natural oils and compatibilizers, the reproducibility of these effects under real processing conditions is still inconsistent across authors. One of the key directions is the optimization of loading levels in TPS/polyester systems and techniques for incorporating oils into polymeric matrices, with a particular focus on the development of different advanced lubrication methods such as nanoencapsulation, which improves materials’ properties and ensures these are maintained over time, but the mechanisms governing their release kinetics and long-term interactions within these matrices require deeper investigation.

Although substantial progress has been made in developing bioplastics, most formulations remain confined to laboratory-scale demonstrations [232]. Therefore, it is essential to improve the methods used to add active compounds to achieve greater efficiency in the mechanical and thermal properties of the materials. However, due to the versatility and lower cost of fossil-based plastics, bioplastics are produced in limited quantities. Further research aimed towards improving these aspects, without a significant increase in their production cost, is therefore required to reduce the competitiveness gap between conventional plastics and renewable polymers [74], given that bioplastics have greater potential to offer environmental sustainability and a lower carbon footprint, and to contribute to the reduction in plastic waste accumulation [233].

Selective compatibilization approaches could improve phase dispersion and interfacial adhesion [234], yet most published work evaluates these improvements only qualitatively or under narrow experimental conditions. Future studies should systematically correlate morphology with mechanical, thermal, and functional performance to build predictive structure–property relationships.

Furthermore, the incorporation of advanced fabrication techniques, such as 3D printing, represents an emerging trend with great potential for the customized and scalable production of composites with complex geometries and properties tailored to specific applications [235], but their impact on TPS degradation kinetics and polyester crystallization has been insufficiently explored. In the field of nanotechnology, the development of nanocomposites based on TPS/polyester blends with fillers or nano-reinforcements is gaining relevance, especially with the incorporation of nanoparticles with antimicrobial properties, such as zinc oxide and copper oxide [236,237].

### Life Cycle Assessment (LCA) Considerations for TPS/Polyester Blends

**Figure 8:**
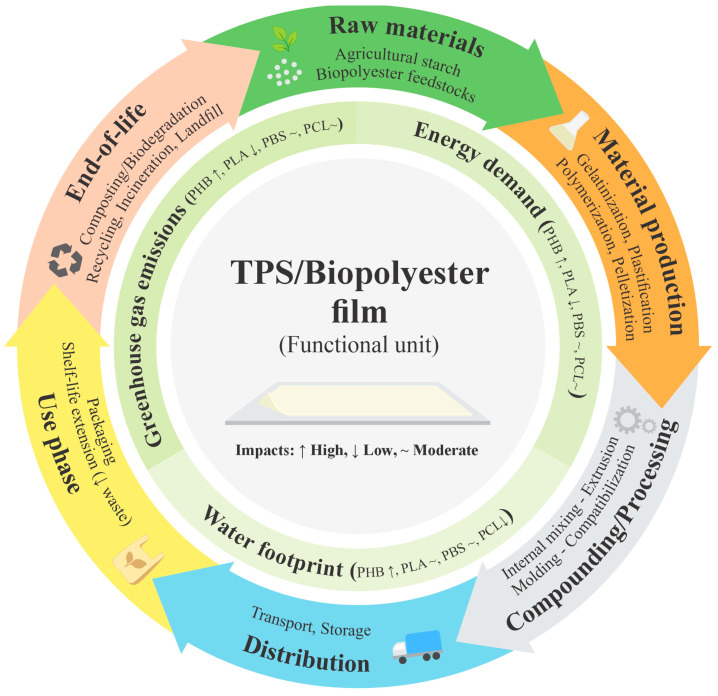
Life cycle assessment (LCA) framework for TPS/biopolyester blends.

Although TPS blends with biodegradable polyesters such as PHB, PLA, PBS, and PCL have been thoroughly studied from a structural, thermal, and mechanical perspective, there is a significant gap in their environmental assessment using LCA [238]. This type of assessment is key to determining whether functional improvements achieved through compatibilization and the use of natural additives actually lead to sustainable benefits when considering aspects such as energy consumption [239], greenhouse gas emissions, and potential recyclability or compostability [240]. These interlinked factors can be better understood through an LCA framework of TPS/biopolyester blends, as illustrated in Figure 8.

Some preliminary studies have indicated that blends with PLA could reduce the environmental impact of pure PLA [241]. However, in the case of TPS/PHB, the benefits are more uncertain due to the high energy demand of PHB production [242]. The use of synthetic compatibilizers or essential oils should also be carefully evaluated, as their incorporation may alter the environmentally expected balances [243].

A clear direction for future research is to integrate LCA approaches from the blend design phase, especially when scaling up to industrial applications, which would help prioritize formulations not only for performance but also for their overall environmental viability. This will allow early identification of the main impacts throughout the entire life cycle of the material, facilitating the selection of processes and additives that optimize both the functional properties and the overall sustainability of the product.

## 6. Conclusions

This review provides a comprehensive overview of recent advances in TPS/biopolyesters blends, with a particular emphasis on compatibilization strategies and the incorporation of bioactive functionalities. The literature consistently shows that the intrinsic incompatibility between hydrophilic TPS and hydrophobic polyesters remains a key challenge, directly affecting morphology, interfacial adhesion, and overall performance. Chemical modification of starch, reactive compatibilization, and the use of bio-based additives such as organic acids, oils, and phenolic compounds have proven effective in improving phase dispersion and tailoring thermal and mechanical behavior.

In addition, the integration of bioactive agents introduces multifunctionality to TPS/polyester systems, enabling antimicrobial and antioxidant responses while simultaneously influencing structural and physical properties. However, the reported effects strongly depend on additive type, concentration, and processing conditions, highlighting the need for systematic studies that link formulation, structure, and performance.

Despite significant progress, several challenges remain, including long-term stability, scalability of progressing routes, and the lack of standardized methodologies for evaluating biodegradation and bioactivity. Future research should focus on optimizing compatibilization efficiency using sustainable approaches, improving structure–property relationships, and assessing environmental performance through life cycle analysis. Addressing these aspects will be essential to advance TPS/polyester blends toward high-performance, sustainable polymeric materials suitable for a wide range of applications.

## Figures and Tables

**Table 1:** Thermal, mechanical, and physical properties of common biopolyesters.

Polymer	PHB ^1^	PLA ^2^	PBS ^3^	PCL ^4^	References
Crystallization Temperature (°C)	110	110–130	75–83	<45	[79,80,81,82]
Melting Temperature (°C)	160–180	175	115	55–65	[70,74,80,83,84,85,86]
Glass Transition Temperature (°C)	2–5	60	−22	−65	[70,74,80,83,84,86]
Elongation at Break (%)	4	3	210	20–1000	[70,84,85,87,88]
Young Modulus (GPa)	2–4	3.4	0.3–0.5	0.21–0.44	[81,83,85,87,88]
Tensile Stress (MPa)	40	60	36	4–785	[74,84,85,87,88]
Density (g/cm^3^)	1.2	1.2–1.3	1.2	1.0–1.2	[70,74,84,85,88]
Biodegradable	Si	Si	Si	Si	[74,80,84,85,86]

^1^ Polyhydroxybutyrate ^2^ Polylactic Acid ^3^ Polybutylene Succinate ^4^ Polycaprolactone.

**Table 2:** Processing methods and property responses of non-compatibilized TPS/biodegradable polyester blends.

Blend	Processing Method	Composition (wt%)			Response				References
		TPS	Polyester	Other	MP ^1^	TP ^2^	DP ^3^	SP ^4^	
TPS/PHB	Internal Mixing	99	1	-	↑	↑	↑	-	[92]
		97	3	-	↑	↑	↑	-	
		95	5	-	↑	↑	↑	-	
		93	7	-	↑	↑	↑	-	
	Compression Molding	10	90	-	↓	↑	-	↓	[112]
		20	80	-	↓	↑	-	↓	
		30	70	-	↓	↓	-	↓	
		40	60	-	↓	↑	-	↓	
		50	50	-	↓	↑	-	↓	
TPS/PLA	Extrusion	40.6	59.4	-	↓	-	↑	↓	[113]
		27	73	-	↓	-	↓	↓	[114]
		43	57	-	↓	-	↓	↓	
		60	40	-	↓	-	↑	↓	
	Internal Mixing	30	70	-	↓	↑	↑	↓	[115]
PLA/TPS/PHB	Extrusion	50	5	45	↓	↑	-	-	[116]
		50	10	40	↑	↑	-	-	
		50	15	35	↑	↑	-	-	
		50	20	30	↑	↑	-	-	
TPS/PBS	Extrusion	80	20	-	↓	↑	↑	↓	[117]
		60	40	-	↓	↑	↑	↓	
		40	60	-	↑	↑	↑	↓	
		20	80	-	↑	↑	↑	↑	
		80 ^5^	20	-	↓	↑	↑	↓	
		60 ^5^	40	-	↑	↑	↑	↑	
		40 ^5^	60	-	↑	↑	↑	↑	
		20 ^5^	80	-	↑	↑	↑	↑	
TPS/PCL	Extrusion	40	60	-	↑	↑	↓	↓	[118]
		50	50	-	↑	↑	↓	↓	
		60	40	-	↓	↓	↑	↓	
		80	20	-	↓	↑	↑	↓	[119]
		80	20	-	↑	↑	↑	↓	

^1^ Mechanical properties ^2^ Thermal properties ^3^ Degradative properties ^4^ Morphological properties ^5^ Waxy starch TPS. Arrows indicate qualitative changes in the reported properties relative to the corresponding neat polymer matrix (↑ increase, ↓ decrease, - not reported). For PLA/TPS/PHB blends, the polyester content corresponds to PHB, and PLA is reported under the “Other” column.

**Table 3:** Effects of different compatibilizers on the properties of TPS/biopolyester blends.

Blend	Compatibilizer	Type	wt% *	Effect on the Blend	Enhanced Properties	References
TPS/PHB	PHB-g-AA (acrylic acid graft)	Functionalized copolymer	Up to 50	Ester–starch bond formation, enhanced interfacial adhesion, lower viscosity.	Mechanical, Processability, Water resistance.	[127]
	PVAc (starch graft)	Modified starch	10–50	Compatibility and thermal stability improvement.	Tenacity, Thermal, Miscibility.	[124]
	Plasma (Aire/SF6)	Surface treatment	10–30	Surface rugosity increase, compatibility improvement.	Mechanical, Interfacial adhesion.	[128]
TPS/PLA	Starch-graft-PLA	Grafted copolymer	2.5–5	Morphology improvement (enhanced dispersion, reduction in PLA spherulites).	Barrier (O_2_), Thermal, Mechanical, Transparency.	[129]
	CA	Plasticizer/Crosslinker	5	Partial starch esterification, improved PLA-TPS adhesion.	Thermal, Mechanical, Biodegradability, Barrier.	[130]
	Zeolite 5A + Biaxial stretching	Physical inorganic filler	1	Reduction in dispersed phase of higher crystallinity and chain orientation.	Mechanical (↑100%), Barrier (O_2_ ↑90%, H_2_O ↑65%).	[113]
	PCL-g-MA	Grafted copolymer	2.5–5	Improvement in interfacial adhesion, reduction in phase separation, morphology control.	Barrier (O_2_), Mechanical, Transparency.	[131]
TPS/PBS	RPBS (PBS + NCO)	Grafted copolymer	10	Improvement in interfacial compatibility and adhesion, reduction in water absorption.	Mechanical, Hydrophobicity, Morphology, Water absorption.	[132]
	CA	Crosslinker	0.1	Decrease in interfacial tension, increase in crystallinity and opacity.	Mechanical, Morphology, Cost, Biodegradability.	[133]
	Maleic anhydride/Tartaric acid	Reactive compatibilizer	1.2–4	Formation of grafted copolymers, improved interfacial adhesion.	Mechanical, Morphology, Structural.	[134]
	Epoxy resin–CHG	Reactive compatibilizer	0.5–5	Reaction between epoxy and amino groups, interfacial adhesion, cohesion and morphology.	Mechanical, Thermal, Hydrophobicity.	[135]

* The reported wt% corresponds to the content of the compatibilizer or additive used in the TPS/biopolyester blend, as reported in the original studies.

**Table 4:** Esterifying properties of citric acid vs. stearic acid.

	Citric Acid	Stearic Acid	References
Reaction type	Crosslinking/Esterification	Esterification	[139,140,146,148]
Degree of substitution	From 0.1 to 0.3	Up to ~0.3 (dry), or ~0.07 (acuous)	[140,143,146,147]
Crystallinity	Decreases	Decreases strongly	[140,148]
Hydrophobicity increase	Moderate	High	[138,140,146,149]
Thermal stability	Enhanced	Enhanced	[139,148]
Mechanical properties	Increases tensile stress	Decreases flexibility, increases stiffness	[139,148]
Emulsion stabilization	Effective	Moderate–Low	[140,149]
Compatibility with biopolyesters	Improves blends phase dispersion	High interfacial compatibility due to hydrophobicity	[143,146]

**Table 5:** Effect of oily and phenolic compounds in biopolyesters blends.

Oil Type	Methods	Oil Content *	Blank	Response	References
Maleinized linseed/Epoxidized fatty acid ester	Extrusion	5–20%	PHB	The oils improved elongation, tensile strength, toughness, and decomposition point. A decrease in T_m_ was obtained.	[176]
Maleinized lindseed/Maleinized soybean	Extrusion/Injection molding	5–20%	PHB	Linseed oil was more effective than soybean oil at improving mechanical properties. Both oils were able to increase the decomposition point of PHB.	[79,177]
Epoxidized soybean	Catalysis	-	Porous PHB	The oil improved the toughness and stiffness, while maintaining the porosity of the PHB. Transparency was not compromised.	[178]
Citronella/Cinnamon	Casting	30%	PHB	Showed effectiveness in all microorganisms of the analysis. Provided greater flexibility to PHB.	[46]
Melaleuca/Cinnamon	Casting	30%	PHB	It was able to inhibit all microorganisms tested. Increased flexibility.	[46]
Melaleuca/Citronella	Casting	30%	PHB	There was no evidence of anti-microbial activity. The blends with PHB had low UV radiation transmission.	[46]
Cinnamaldehyde	Casting	5%	PLA/PHB	Improved mechanical and active properties were evidenced. The proliferation of microorganisms in a piece of salmon was inhibited.	[64]
Clove	Immersion	-	PHB/BC	Reduced growth of microorganisms by 65%. Improved thermal properties and degradation temperature.	[179]
Eugenol	Casting	20–40%	PHB	Increase in crystallinity, antimicrobial activity, reduction in mechanical properties.	[180]
Eugenol	Extrusion	2.5–3.5%	PHB/TPS/OMMT	Increase in melting temperature and reduction in elastic modulus. Antioxidant and antimicrobial activity.	[35]
Epoxidized soybean	Extrusion	10–20%	TPS/PBAT	Improved elongation at break (>600%). Preservation of thermal and barrier properties with increased compatibility.	[181]
Epoxidized linseed	Extrusion	1 phr	TPS/PBAT	Increased elongation at break (~94%), improved chain mobility; useful as a plasticizer and compatibilizer.	[182]
Sunflower (fried)	Extrusion	5–10%	TPS/PBS	Reduction in the melting temperature of TPS. Improved homogeneity and thermal stability. Useful as a low-cost sustainable additive.	[183]

* (wt% relative to polymer matrix). When no value is reported (-), the corresponding concentration was not specified in the original study.

## Data Availability

The original contributions presented in this study are included in the article. Further inquiries can be directed to the corresponding author.
